# Compartment-Specific Endometrial Gene Analysis in Infertile Women with Thin *Versus* Normal Endometrium on Oocyte Retrieval Day: Insights from a Cross-Sectional Study

**DOI:** 10.5935/1518-0557.20250193

**Published:** 2026

**Authors:** Pavarit Humart, Kittima Tungprasertpol, Supatra Klaymook, Somsin Petyim

**Affiliations:** 1 Reproductive Biology and Infertility Unit (RBU), Department of Obstetrics and Gynecology, Faculty of Medicine Siriraj Hospital, Mahidol University, Bangkok, 10700, Thailand; 2 Center of Reproductive Genetics and Stem cell Biology (CRGSCB), Department of Obstetrics and Gynecology, Faculty of Medicine Siriraj Hospital, Mahidol University, Bangkok, Thailand

**Keywords:** gene expression, endometrial thickness, thin endometrium, infertile women

## Abstract

**Objective:**

Thin endometrium is an increasingly recognized challenge in infertility treatment. This study aimed to compare endometrial gene expression between women with thin and normal endometrial thickness, and also to identify ovarian stimulation-related factors associated with thin endometrium.

**Methods:**

Endometrial tissue samples were collected from infertile women undergoing controlled ovarian stimulation, including 10 samples with thin endometrium (less than 7 millimeters) and 10 with normal thickness (7 millimeters or more) on the day of egg retrieval. Gene expression was analyzed using quantitative real-time polymerase chain reaction.

**Results:**

The relative expression of *LGR5* in the luminal epithelium was slightly higher in the thin group, while *POU5F1* expression in the stromal area was higher in the normal group. In the perivascular region, *PDGFRB* and *SUSD2* were more highly expressed in the normal group, with *SUSD2* showing a statistically significant difference (*p*=0.034). In addition, under higher serum estradiol levels, both *POU5F1* and *LGR5* showed greater expression in the normal group compared to the thin group with lower estradiol levels.

**Conclusions:**

Reduced *SUSD2* expression in the perivascular region, a marker of endometrial stem and progenitor cells, may contribute to impaired endometrial growth. Serum estradiol levels also appear to influence gene expression within different endometrial compartments. These findings may offer insights into new therapeutic strategies for managing thin endometrium.

## INTRODUCTION

Assisted Reproductive Techniques (ART) have undergone significant developments in recent years. Although the ART process has advanced significantly, there are still some problems reducing the succession rate that need to be resolved. Successful implantation requires high-quality embryos and good-quality of endometrium (Diedrich *et al.*, 2007). In a normal menstrual cycle. endometrial alterations are regulated by cyclic hormonal changes in the Hypothalamic-Pituitary-Ovarian (HPO) axis (Qin *et al.*, 2021). The quality and thickness of the endometrium are also influenced by hormones produced through ovarian steroidogenesis. The capacity of the endometrial to proliferate and nurture an embryo is critical for implantation and successful pregnancy outcomes.

The receptive endometrium is defined as a healthy uterine environment containing the transformation of endometrial cells into decidual cells, which are appropriate for the implantation of blastocysts and the growth of the placenta (Zenclussen & Hämmerling, 2015). Endometrial assessment is a crucial part of ART process. It helps determine endometrial receptivity, which is important for successful implantation and pregnancy (Diedrich *et al.*, 2007). There are various methods of endometrial assessment that are used, such as endometrial thickness (ET), pattern, volume, Doppler studies, or endometrial receptivity analysis (Liu *et al.*, 2019). Among these methods, transvaginal ultrasonography (TVS) is the ideal noninvasive tool to evaluate the endometrium. It is safe, takes little time, and is easy to perform. Endometrial thickness is an important parameter that is routinely measured during in vitro fertilization (IVF) and intracytoplasmic sperm injection (ICSI) cycles. ET is directly correlated with increased circulating estrogen levels, which play a crucial role in the preparation of the endometrium for implantation (Zenclussen & Hämmerling, 2015; Liu *et al.*, 2019).

Endometrial thickness is related to endometrial receptivity and has been considered a prognostic factor for embryo transfers during IVF/ICSI treatment. Endometrial thickness of 8-14 mm is considered optimal for the success IVF and ICSI (Kasius *et al.*, 2014). The thin endometrium (TEM) is one of the potential problems of ART. Several studies have demonstrated that an ET of at least 7 mm increases the pregnancy rate and reduces the risk of miscarriage compared to an ET below 7 mm (Kumbak *et al.*, 2009; Wu *et al.*, 2014; Safari *et al.*, 2024). Data from a meta-analysis revealed that ET of 7 mm or less resulted in a lower pregnancy rate, with an odds ratio of 0.42 (95% CI 0.27,0.67) (Kasius *et al.*, 2014). Studies have reported that an ET less than 7 mm occurs with an incidence of 0.7%-2.5% in fresh IVF cycles (Shufaro *et al.*, 2008; Wu *et al.*, 2014; Bu & Sun, 2015). Also, data from the Reproductive Biology and Infertility Unit of Siriraj Hospital, the incidence of TEM in the controlled ovarian stimulation (COS) cycle was 2%. Recent studies have shown that frozen embryo transfer in cases of thin endometrium is associated with low birthweight infants, underscoring the importance of endometrial thickness in IVF outcomes (He *et al.*, 2022a, 2022b; Zheng *et al.*, 2022; Tang *et al.*, 2023; Sun *et al.*, 2024).

A TEM has become an increasingly common issue during infertility treatments, particularly in artificial endometrial preparation. The causes of this condition are varied and can include idiopathic factors, iatrogenic effects, underlying medical conditions, and the use of certain medications. These contributing factors include Asherman syndrome, prior endometrial surgery or curettage, pelvic radiation, clomiphene citrate, endometritis, septic abortion, Müllerian anomalies, a hypoestrogenic state, and the use of progestins or androgens (Liu *et al.*, 2019). However, even after addressing the causes of a thin endometrium such as through hysteroscopic adhesiolysis in cases of uterine synechiae many patients fail to achieve full endometrial thickness following endometrial preparation. During ovarian stimulation, optimal endometrial thickness is expected due to the high levels of estrogen produced by multiple follicles. Yet, in some cases, normal endometrial thickness is not achieved, and the exact etiology of this endometrial growth retardation, particularly during ovarian stimulation, remains unclear.

The endometrium is composed of various cell types with distinct functions, located in different compartments including the basalis, luminal epithelium, stroma, and perivascular regions (Beier & Beier-Hellwig, 1998; Diedrich *et al.*, 2007; Zenclussen & Hämmerling, 2015). Each of these cells contributes to specific functions within their respective locations. With molecular basis, gene expression that regulates the proliferation and transformation of endometrial cells in the each compartment is partly controlled in a spatial temporal manner (Alberts *et al.*, 2002). Interestingly, several genes are expressed at specific times and locations within the endometrium. According to studies by Tempest *et al.* (2018; 2022) and Cousins *et al.* (2021), key endometrial genes are highly expressed in different regions, such as Fucosyltransferase 4 (*FUT4)*, Cadherin 2 (*CDH2)*, and SRY box transcription factor 9 (*SOX9)* in the basalis; Leucine rich repeat containing G protein coupled receptor 5 (*LGR5)* in the luminal epithelium; POU class 5 homeobox 1 (*POU5F1)* in the stroma; and Platelet derived growth factor receptor beta (*PDGFRB)*, Sushi domain containing 2 (*SUSD2)*, and Melanoma cell adhesion molecule (*MCAM)* in the perivascular region.

In cases of ovarian stimulation, many patients fail to respond adequately to hormonal treatment, resulting in a thin endometrium. This study aims to analyze gene expression in different components of the endometrium in cases of thin endometrium (ET < 7mm) compared to normal endometrial thickness (ET ≥ 7 mm) and to identify any factors related to ovarian stimulation that may be associated with a thin endometrium. The findings may provide insights into the underlying causes of thin endometrium and offer implications for infertility treatment in affected women. By understanding the genetic factors contributing to a thin endometrium, it may be possible to develop more effective treatment strategies, ultimately improving the likelihood of successful implantation and pregnancy.

## MATERIALS AND METHODS

### Endometrial sample collection

This cross-sectional study was conducted at the Department of Obstetrics and Gynecology, Faculty of Medicine Siriraj Hospital, Mahidol University. The study protocol was approved by the Siriraj Institutional Review Board, Faculty of Medicine Siriraj Hospital, Mahidol University, with a certificate of approval no. Si 183/2022 before enrolling participants.

Women aged 18 to 45 years, diagnosed with infertility and undergoing controlled ovarian stimulation (COS) using a gonadotropin releasing hormone GnRH antagonist protocol, were recruited from the Reproductive Biology and Infertility Unit, Department of Obstetrics and Gynecology, Siriraj Hospital for planned frozen-thawed embryo transfer. Prior to enrollment, all participants underwent transvaginal ultrasonography (TVS) with saline infusion sonohysterography (SIS) to evaluate for endometrial, myometrial, and adnexal pathologies. Exclusion criteria included the presence of endometrial polyps, submucous myomas, uterine synechiae, congenital uterine anomalies, cervical stenosis, cervical cancer, adenomyosis, endometriomas, or hydrosalpinx. Additional exclusions were prior uterine radiation, current genital tract or active sexually transmitted infections, bleeding disorders, a history of endometrial hyperplasia or malignancy, and current use of clomiphene citrate, aromatase inhibitors, progestins, or androgens. Patients who had undergone intrauterine surgery or uterine curettage within the past three months were also excluded. The researcher provided a clear explanation of the study’s objectives, the research process, and the methods of data collection. Additionally, the researcher ensured that patients fully understood the confidentiality measures in place to protect their privacy. Patients were informed about the study procedures, enabling them to make an informed decision regarding their participation.

Patients then underwent the COS, during which the dosage and duration of drug intake were determined by the treating physician. On the day of oocyte retrieval, those meeting the study’s inclusion and exclusion criteria were invited to participate. They received a detailed document explaining the research and were required to sign a consent form before proceeding with oocyte collection. Patients who agreed to participate were identified as research participants, with a sticker placed on the front of their medical history file at the Reproductive Biology and Infertility Unit.

Before starting the oocyte retrieval process, the same research assistant will measure the endometrial thickness using TVS for all participants. Additionally, a 10 ml blood sample will be collected to measure estradiol (E2) levels. The attending nurse will administer parenteral sedatives and pain relievers. After the oocyte retrieval process, another research assistant, who is blinded to the participants’ ET, will perform an endometrial biopsy. This same assistant will collect endometrial samples from all participants. Once the tissue samples are extracted, they will be placed in a normal saline solution. The co-investigators will then transfer the endometrial tissue into a prepared solution tube, which will be promptly delivered to the laboratory for gene expression analysis. Participants will be excluded from the study if the collected endometrial sample is insufficient for quantitative real-time polymerase chain reaction (qPCR) analysis or if endometrial sample collection is unsuccessful.

### Histology test and Immunofluorescence

Endometrial tissue samples were embedded in optimal cutting temperature (OCT) compound, which then underwent frozen sectioning on a microtome-cryostat. Then tissues were sectioned into 5 µm thick slices and stained with hematoxylin and eosin (H&E) for histological examination. Stained sections were examined under a microscope to assess tissue morphology. For immunofluorescence analysis, subsequent to embedding the endometrial samples in OCT compound, tissues were subjected to frozen sectioning via a microtome-cryostat. Tissue was cut into 10 microns and placed onto a glass slide to perform immunofluorescence staining. Tissue was fixed with 4% paraformaldehyde for 15 min, followed by washes with PBS supplemented with 0.1% Tween 20 (PBST) three times, and then blocked with 1% bovine serum albumin in PBST for 1 hr at room temperature. Subsequently, tissue was incubated with primary antibody, composed of mouse anti-pan-CK (1:100, ab86734, Abcam, Cambridge, UK) and rabbit anti-vimentin antibody EPR3776 (2 µg/ml, ab92547, Abcam), overnight at 4°C. Tissues were then washed with PBST, followed by secondary antibody staining, including goat anti-mouse IgG1, Alexa Fluor™ 488 (A21121, Thermo Fisher Scientific, MA, USA), and goat anti-rabbit IgG (H+L), Alexa Fluor™ 555 (A21429, Thermo Fisher Scientific), at room temperature for 1 hr. Tissues were finally mounted with antifade mounting medium with DAPI (Vector Laboratories, CA, US) in the dark and imaged by using a confocal microscope (Nikon, Shinagawa-ku, Tokyo).

### Gene expression analysis

Endometrial gene expression analysis was carried out at the Center of Reproductive Genetics and Stem Cell Biology, Department of Obstetrics and Gynecology, Faculty of Medicine, Siriraj Hospital. The primers for *FUT4, CDH2, SOX9, LGR5, POU5F1, PDGFRB, SUSD2*, and *MCAM* genes were designed based on the PubMed gene database and using the primer-BLAST tool available on the NCBI database. The primers for the genes of interest (Table 1) were ordered from Pacific Science CO., LTD, Thailand.

**Table 1:** Primer of interested gene.

Gene	Forward Primer	Reverse Primer	Annealing temp. (°C)	Amplicon size (bp)
*FUT4*	5'- AGGGGGTTCTTCCTCACCTT -3'	5'- ATATGGCCTGTGGCAGATGG -3'	59	105
*CDH2*	5'- GACGGTTCGCCATCCAGAC -3'	5'- TCGATTGGTTTGACCACGG -3'	56	67
*SOX9*	5'- AGCGAACGCACATCAAGAC -3'	5'- GCTGTAGTGTGGGAGGTTGAA -3'	58	110
*LGR5*	5'- CACCTCCTACCTAGACCTCAGT -3'	5'- CGCAAGACGTAACTCCTCCAG -3'	61	94
*POU5F1*	5’- ACATCAAAGCTCTGCAGAAAGAACT-3’	5’-CTGAATACCTTCCCAAATAGAACCC -3’	56	127
*PDGFRB*	5'- TGGTGCTCACCATCATCTCC -3'	5'- CACCTTCCATCGGATCTCGTAA -3'	59	80
*SUSD2*	5'- CAGCTACCAGAGATGGCGAGTGG -3'	5'- CCTCTCGGAAGTCATCGCTCAGG -3'	61	134
*MCAM*	5'- ATCGCTGCTGAGTGAACCACAG -3'	5'- CTACTCTCTGCCTCACAGGTCA -3'	58	123
*ACTB*	5'- ATGTGGCCGAGGACTTTGATT -3'	5'- AGTGGGGTGGCTTTTAGGATG -3'	58	107

Endometrial tissue samples obtained from endometrial biopsy were washed with normal saline to remove all blood and then frozen in liquid nitrogen, which was kept at -80°C until the RNA extraction. The RNA extraction procedure involved homogenizing the tissue sample using a micropestle and performing RNA extraction using the GenElute^®^ Total RNA Purification Kit (Sigma-Aldrich Co. LLC, MO, USA) following the manufacturer’s protocol. For cDNA synthesis, the iScript^®^ Reverse Transcription Supermix (Bio-rad Laboratories, Inc., CA, USA) was used according to the manufacturer’s protocol.

To perform qPCR analysis, the LightCycler^®^ 480 system and the Roche SYBR Green I Master kit (Roche, Basel, Switzerland) were used to evaluate mRNA expression according to the manufacturer’s protocol. The results from qPCR were cycle threshold (CT) and gene expression data were analyzed using the 2^-∆∆Ct^ quantitative method to estimate relative fold change values. Following each qPCR run, the melting curves were analyzed to confirm the specificity of the primers, which was indicated by the presence of a single band and no artifacts of primer-dimer.

### Statistical analysis

The sample size for this study was calculated based on a pilot study involving 10 participants, with a dropout rate of 10%, a β level of 0.02, and an α level of 0.05. Twenty participants were required (10 participants in each group). Data were analyzed using SPSS version 26. To assess the normality of continuous data, Shapiro-Wilk or histogram plots were employed. Continuous data was analyzed using either unpaired t-test or the Mann-Whitney U test. On the other hand, categorical data was analyzed using the Chi-squared test or Fisher’s exact test. Unpaired t-test was used to identify significant differences between the means of two groups. A *p*-value < 0.05 was considered statistically significant.

## RESULTS

In the study, twenty participants were recruited between May 2022 and February 2023 and divided into two groups: ten participants in the thin endometrium (TEM) group and ten participants in the normal endometrium (NEM) group. Each participant provided an endometrial sample on the day of oocyte retrieval, which was used to examine the expression of various genes at different compartments within the endometrium. Descriptive statistics were used to analyze the baseline characteristics of the participants. The mean age of the TEM group was 38.5±2.71 years, while the mean age of the NEM group was 37.5±4.48 years. The major causes of infertility were male factor infertility and unexplained infertility, with 35% of participants having a history of endometrial surgery. Each participant received individualized ovarian stimulation using gonadotropins, including human menopausal gonadotropin (hMG) and recombinant follicle-stimulating hormone (rFSH). The mean total dose of gonadotropin was 2,485±94.59 IU in the TEM group and 2,355±153.56 IU in the NEM group. Human chorionic gonadotropin (hCG) was used as the ovulation trigger and for final oocyte maturation in 55% of cases. The mean endometrial thickness was 6.1±0.34 mm in the TEM group and 12.2±0.49 mm in the NEM group. The mean estradiol (E2) level on the day of oocyte retrieval, an indicator of ovarian function and endometrial stimulation, was 1,254.1±223.9 pg/ml in the TEM group and 1,108.5±128.1 pg/ml in the NEM group (Table 2).

**Table 2:** Descriptive statistics of the baseline characteristics.

Parameters	Statistic	Thin endometrium (n=10)	Normal endometrium (n=10)	p-value
Age (y)	Mean (SD)	38.47 (2.71)	37.51 (4.48)	0.571
BMI (kg/m^2^)	Mean (SD)	22.45 (3.77)	23.11 (2.91)	0.881
Cycle length (day)	Mean (SD)	28.3 (1.7)	30.0 (2.4)	0.081
AFC	Mean (SD)	7.50 (1.29)	9.80 (1.30)	0.218
AMH (ng/ml)	Mean (SD)	1.75 (0.38)	2.14 (0.41)	0.353
Cause of infertility -Tubal factor -Male factor -Unexplained	N (%)	1 (10) 3 (30) 6 (60)	1 (10) 5 (50) 4 (40)	0.809
Previous endometrial surgery or uterine curettage	N (%)	4 (40)	3 (30)	1.000
Type of gonadotropin -hMG -rFSH	N (%)	6 (60) 4 (40)	5 (50) 5 (50)	1.000
Dose of gonadotropin (IU)	Mean (SD)	2485 (94.59)	2355 (153.56)	0.579
Type of oocyte triggering - hCG - GnRHa - hCG+GnRHa	N (%)	5 (50) 1 (10) 4 (40)	6 (60) 1 (10) 3 (30)	1.000
Endometrial thickness (mm)	Mean (SD)	6.10 (0.34)	12.18 (0.49)	0.000
Number of follicles more than 17 mm on day of oocyte retrieval	Mean (SD)	6.80 (0.69)	8.40 (0.91)	0.275
Estradiol level on day of oocyte retrieval (pg/ml) ≤ 1000 > 1000	Mean (SD) N (%)	1254.10 (223.89) 5 (50) 5 (50)	1108.50 (128.09) 6 (60) 4 (40)	0.853

BMI = body mass index, AFC = antral follicle count, AMH = Anti-Mullerian Hormone, hMG = human menopausal gonadotrophin, rFSH = recombinant follicle-stimulating hormone, LH = luteinizing hormone, hCG = human chorionic gonadotropin, GnRHa = gonadotropin releasing hormone, MII = Metaphase II, MI = Metaphase I, GV = germinal vesicle.

### Histological study

**Figure 1:**
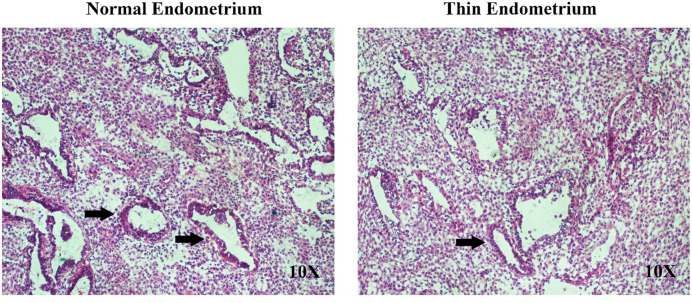
Histology of endometrial tissues was characterized by hematoxylin and eosin (H&E) staining, which showed endometrial glands (black arrows) and stromal cells in both normal and thin endometrium groups.

**Figure 2:**
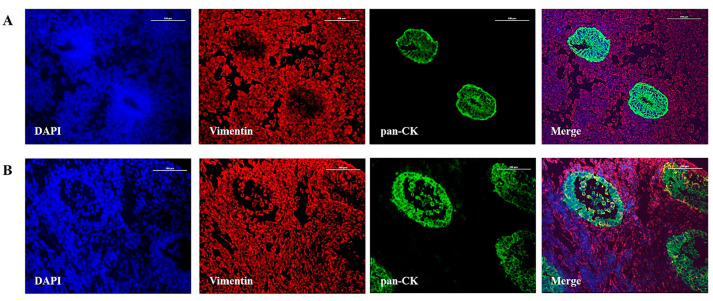
Immunofluorescence staining of endometrial tissues showed pan-cytokeratin as an epithelial marker (green), vimentin as a stromal marker (red), and DAPI (blue) in both normal (A) and thin (B) endometrium groups. Scale bar: 100 µm.

H&E stained sections of endometrial biopsy tissues demonstrate variation in size and shape of endometrial glands and present stromal cells within both normal and thin endometrium groups (Figure 1). For immunofluorescence staining, endometrial tissue samples in both normal and thin endometrium groups exhibited positivity of epithelial marker (cytokeratin) and stromal marker (vimentin), which are presented in Figure 2.

### qPCR analysis

**Figure 3:**
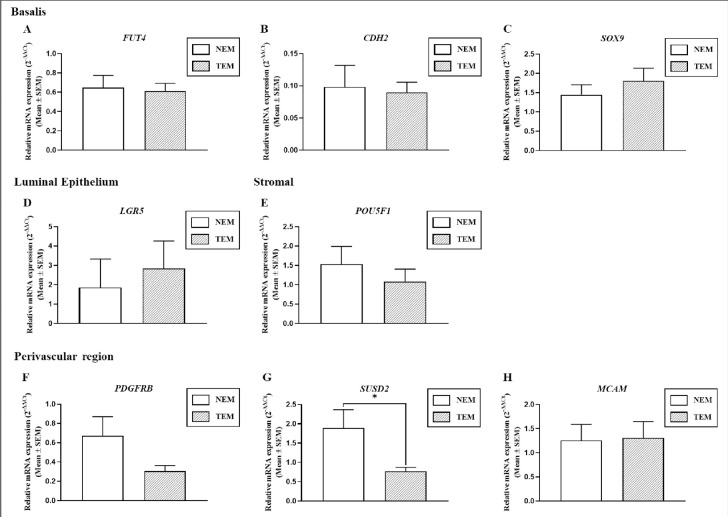
Comparative expression of endometrial genes between thin endometrium (TEM) and normal endometrium (NEM) groups. The results are relative mRNA expression as 2^-∆∆ct^ (Mean±SEM). The gene of interests are divided as endometrial gene expression at different locations, including *FUT4, CDH2*, and *SOX9* at the basalis; *LGR5* at the luminal epithelium; *POU5F1* at the stroma; and *PDGFRB, SUSD2*, and *MCAM* at the perivascular region.

Endometrial tissues were extracted and converted into cDNA for relative gene expression analysis. Data from the qPCR analysis showed a significant difference in the relative fold change (2^-∆∆Ct^) of endometrial gene expression in the perivascular region between the TEM group and the NEM group. In the basalis region, the expression levels of genes such as *FUT4, CDH2*, and *SOX9* were similar between the two groups (Figure 3; A-C). The relative expression of *LGR5* in the luminal epithelial region was slightly higher in the TEM group compared to the NEM group (Figure 3D). In contrast, the expression of *POU5F1* in the stromal region was slightly higher in the NEM group compared to the TEM group (Figure 3E). In the perivascular region, the expression of *PDGFRB* was higher in the NEM group, although the difference was not statistically significant compared to the TEM group (Figure 3F). The relative expression of *SUSD2* was significantly higher in the NEM group than in the TEM group (*p*-value = 0.03), as shown in Figure 3G. *MCAM* gene expression was similar between the two groups, with no significant difference observed (Figure 3H).

When considering the type of gonadotropin used to induce follicle growth in the ovary, which affects endometrial gene expression at different compartments, the results showed similar expression levels between the hMG group and rFSH group. The luminal epithelial region gene (*LGR5*) presented high relative expression in the rFSH group but no statistical significance when compared with the hMG group (Table 3).

**Table 3:** Type of gonadotropin and endometrial gene expression. The gene expression analysis across different endometrial compartments, based on the type of gonadotropin used.

Gene	Location	Relative mRNA expression (2^-∆∆Ct^) (Mean±SEM)		*p*-value
		hMG (n=11)	rFSH (n=9)	
*FUT4*	Basalis	0.5201±0.0714	0.7638±0.1298	0.1012
*CDH2*	Basalis	0.0968±0.0275	0.0907±0.0239	0.8715
*SOX9*	Basalis	1.6720±0.2494	1.5670±0.3626	0.8074
*LGR5*	Luminal epithelium	1.4590±0.7707	3.4330±2.0330	0.3407
*POU5F1*	Stroma	1.2880±0.4040	1.3270±0.4000	0.9460
*PDGFRB*	Perivascular	0.4692±0.1404	0.5128±0.1799	0.8482
*SUSD2*	Perivascular	1.1800±0.2308	1.5080±0.5457	0.5609
*MCAM*	Perivascular	1.0530±0.2872	1.5630±0.3730	0.2854

hMG = human menopausal gonadotrophin, rFSH = recombinant follicle-stimulating hormone.

**Figure 4:**
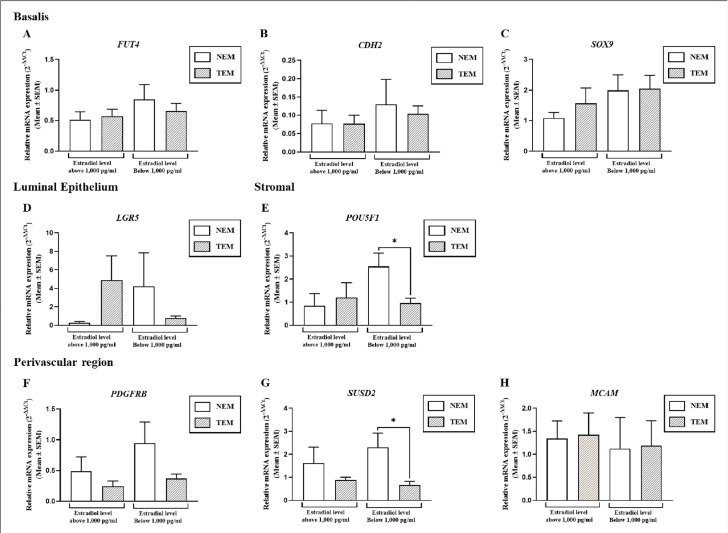
When considering the estradiol level in blood that could affect to pregnancy rate. The comparative of estradiol levels between over 1,000 and below 1,000 pg/ml was determined in relative mRNA expression and also compared between thin endometrial thickness (TEM) and normal endometrial thickness (NEM) groups. The graph was depicted in relative mRNA expression (Mean±SEM) of endometrial gene expression at different locations, including the basalis (A-C); the luminal epithelium (D); the stroma (E); the perivascular region (F-H).

The E2 level in the blood is related to the pregnancy rate in IVF patients. When considering the E2 levels which were divided into two groups (above 1,000 pg/ml and below 1,000 pg/ml) and also compared between TEM and NEM groups. The genes *FUT4* and *CDH2* in the basalis region showed no significant difference among all groups. The results revealed slightly higher expression in the NEM group with E2 levels below 1,000 pg/ml (Figure 4A-B). The *SOX9* gene presented a gradually increased relative gene expression in the TEM group compared to the NEM group in serum E2 levels above 1,000 pg/ml. The gene expression level in the below 1,000 pg/ml group exhibited similar results in both TEM and NEM groups (Figure 4C). The group with E2 above 1,000 pg/ml exhibited high expression of the *LGR5* gene in the TEM group compared with the NEM group. However, in the group with E2 below 1,000 pg/ml, the *LGR5* gene showed contrary results with low expression in the TEM group compared with the NEM group (Figure 4D). For the E2 level below 1,000 pg/ml, the *POU5F1* gene (stromal region) showed significantly higher expression in the NEM group compared with the TEM group. Conversely, in the E2 level above 1,000 pg/ml group, the *POU5F1* gene exhibited slightly higher expression in the TEM group (Figure 4E). In both E2 levels above 1,000 pg/ml and below 1,000 pg/ml, the *PDGFRB* gene exhibited high expression, but no significant difference was observed in the NEM group (Figure 4F). The *SUSD2* gene showed significantly higher expression in the NEM group compared to the TEM group at E2 levels below 1,000 pg/ml (Figure 4G). The *MCAM* gene showed comparable expression levels across all groups (Figure 4H).

**Figure 5:**
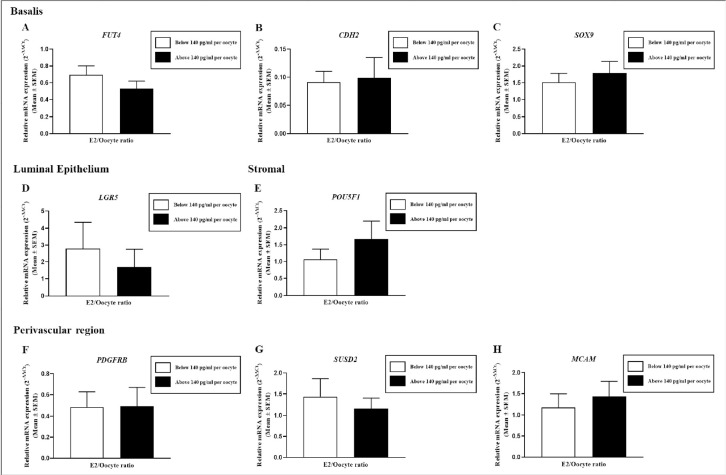
The graph presented the relative mRNA expression level which compares the estradiol/oocyte ratio between above 140 and below 140 pg/ml per oocyte. The data of endometrial genes at different locations, including the basalis (A-C); the luminal epithelium (D); the stroma (E); and the perivascular region (F-H) were displayed as Mean±SEM.

The E2 level and oocyte ratio were considered as additional factors affecting the pregnancy rate. E2-to-oocyte was calculated and divided into two groups, including below 140 pg/ml and above 140 pg/ml per oocyte. The endometrial gene expression level of the basalis region, *CDH2* and *SOX9*, presented slightly high expression in E2-to-oocyte ratio at above 140 pg/ml per oocyte. In contrast, The *FUT4* gene exhibited low expression levels at above 140 pg/ml per oocyte when compared with the E2-to-oocyte ratio at below 140 pg/ml per oocyte (Figure 5A-C). The *LGR5* gene expression in E2-to-oocyte ratio above 140 pg/ml per oocyte showed diminished expression compared to E2-to-oocyte below 140 pg/ml per oocyte (Figure 5D). On the contrary, the *POU5F1* gene displayed a high relative expression level in E2-to-oocyte ratio above 140 pg/ml per oocyte group (Figure 5E). In perivascular region, *PDGFRB* gene, presented comparable relative expression level between both groups (Figure 5F). *SUSD2* gene exhibited higher expression in E2-to-oocyte below 140 pg/ml per oocyte group (Figure 5G). In contrast, the level of *MCAM* gene expression presented slightly high expression in above 140 pg/ml per oocyte when compared with below 140 pg/ml per oocyte (Figure 5H).

## DISCUSSION

The endometrium is a well-accepted factor as a part of IVF success rates as the embryo implantation process requires the healthy endometrium in both quality and quantity (Shufaro *et al.*, 2008; Kumbak *et al.*, 2009; Kasius *et al.*, 2014; Wu *et al.*, 2014; Bu & Sun, 2015; Safari *et al.*, 2024; Sun *et al.*, 2024). Indeed, prior embryo transfers during IVF/ICSI treatment, endometrial thickness is routinely measured to obtain the optimal thickness leading to decision-making for embryo transfer (Safari *et al.*, 2024; Sun *et al.*, 2024). Nowadays, thin endometrial (TEM) lining seems to be a more common problem in IVF treatment and raising a concern to identify the cause of endometrium thickness failure. A TEM may result from previous trauma; however, in many cases, the underlying cause remains unknown. During the ovarian stimulation process, high levels of estrogen typically promote endometrial thickening. When the endometrium itself fails to respond adequately, leading to a thin endometrium, it poses a significant challenge to improving the success rate of implantation and pregnancy. In this study, we define an endometrial thickness (ET) of less than 7 mm as TEM. Several studies have similarly demonstrated that an ET below 7 mm is associated with a decreased pregnancy rate (Kumbak *et al.*, 2009; Kasius *et al.*, 2014; Wu *et al.*, 2014; He *et al.*, 2022a, 2022b; Zheng *et al.*, 2022; Tang *et al.*, 2023; Sun *et al.*, 2024). The objective of this study was to identify factors related to ovarian stimulation that may be associated with a thin endometrium, and to compare the expression of endometrial genes across different components between the TEM and normal endometrial thickness (NEM) groups. By identifying any gene expression differences linked to TEM, these findings could offer insights into the etiology of thin endometrium and suggest potential future treatments for the condition.

In the study, the baseline characteristics of participants, types of gonadotropins used including human menopausal gonadotropin and recombinant follicle-stimulating hormone and the total dose of gonadotropins, as well as the type of ovulation trigger, were comparable between the TEM and NEM groups. However, ET in the TEM group was significantly different from that in the NEM group, while endogenous estradiol (E2) levels on the day of oocyte retrieval were not notably different between the two groups. This might suggest that endometrial thickness may reflect endometrial quality itself. Gene expression analysis, focusing on specific compartments using gene markers predominantly expressed in each region, was conducted to compare TEM and NEM groups. Interestingly, the expression of key endometrial genes in the luminal epithelium (*LGR5*), stroma (*POU5F1*), and basalis (*FUT4, CDH2*, and *SOX9*) showed no significant differences between the two groups. However, gene expression related to the perivascular region (*PDGFRB, SUSD2*, and *MCAM*) was lower in the TEM group compared to the NEM group, particularly for *PDGFRB* and *SUSD2*. These findings suggest that differences in endometrial thickness may be linked to variations in the perivascular region.

Furthermore, we found that the significantly lower expression of *SUSD2* in the thin endometrium group suggests dysregulation in specific cell populations. Previous studies have identified *SUSD2* as a marker for endometrial stem/progenitor cells located around perivascular areas (Murakami *et al.*, 2014; Wyatt *et al.*, 2021; Hong, 2023). Our finding of reduced *SUSD2* expression in the TEM group may indicate a lower number of *SUSD2*-positive endometrial cells or a defect in *SUSD2* gene expression, potentially contributing to the etiology of endometrial growth retardation in this group.

In the endometrium, the major components are the luminal epithelium and stroma, where a balance between mesenchymal and epithelial components is essential. Based on ultrasonographic endometrial assessments, the sonolucent areas in the endometrial stripe are likely related to the stromal part. We hypothesize that an imbalance between stromal and epithelial proliferation could contribute to the reduced endometrial thickness. However, our analysis of gene expression in both components showed a slightly higher expression of stromal genes in the NEM group compared to the TEM group, though this difference did not reach statistically significance. This suggests that the reduction in endometrial thickness may not be primarily caused by, or directly related to, gene expression in the stromal and epithelial components.

In a subgroup analysis based on serum E2 levels on the day of hCG administration, gene expression in the perivascular area was higher in both the TEM and NEM groups, regardless of whether serum E2 levels were low (below 1,000 pg/mL) or high (above 1,000 pg/mL). Notably, differences in *SUSD2* expression reached statistical significance in both groups across high and low serum E2 levels, further confirming that the loss of *SUSD2* expression is associated with a thin endometrium. These findings are consistent with previous studies (Zhu *et al.*, 2021; Lv *et al.*, 2022). Interestingly, we found that in cases of low serum E2, gene expression in the NEM group was significantly higher in both the stromal and luminal components compared to the TEM group. In contrast, in cases of high serum E2, gene expression in the stromal and luminal components was lower in the NEM group than in the TEM group. This suggests that serum E2 levels may influence gene expression in both the stromal and epithelial components. Higher E2 levels may not enhance stromal gene expression as much as they do in epithelial components. It remains unclear, however, whether mesenchymal-to-epithelial transition (MET) is related to endometrial thickness.

In this study, we used a serum E2 level of 1,000 pg/mL as the cut-off for subgroup analysis. Several studies have demonstrated the impact of serum E2 on ART outcomes, with the optimal range of serum E2 levels on the day of hCG administration being between 1,000 and 3,194 pg/mL (Li *et al.*, 2019), and peak serum E2 levels (PSEL) between 1,000 and 4,000 pg/mL influencing pregnancy and implantation rates (Carmona-Ruiz *et al.*, 2010). Our findings on the effect of serum E2 levels on endometrial gene expression are in line with these studies (Carmona-Ruiz *et al.*, 2010; Check *et al.*, 2010; Li *et al.*, 2019).

Previous studies have shown that an E2-to-oocyte ratio between 70-140 pg/mL per oocyte is correlated with pregnancy rates (Aslih *et al.*, 2021), and a positive correlation between the E2-to-oocyte ratio and pregnancy outcomes has been reported (Orvieto *et al.*, 2007). However, in our subgroup analysis of the E2-to-oocyte ratio, we did not observe any significant differences in gene expression levels for any of the genes in both the TEM and NEM groups. Additionally, prior studies have identified gonadotropin and hCG receptors on endometrial cells (La Marca *et al.*, 2005; Tapia-Pizarro *et al.*, 2017), and differences in the type of FSH used have been linked to varying inhibitory effects on endometrial stromal cell proliferation (Kim *et al.*, 2019). In our study, however, we found no correlation between the type of gonadotropin used and gene expression across different endometrial compartments, particularly in genes dominant in stromal and epithelial cells.

In this study, we included participants with a history of previous endometrial surgery or uterine curettage equally across both groups, ensuring comparable baseline characteristics. Only 30-40% of participants in each group had a history of intrauterine basement membrane trauma, with confirmed complete healing after correction. Therefore, the results of this study may highlight gene expression differences in both iatrogenic and idiopathic causes of TEM and NEM groups.

The strength of this study lies in its comparative analysis of gene expression between TEM and NEM during COS, with a focus on distinct endometrial compartments. The exclusion of other endometrial pathologies enables a specific focus on idiopathic cases. This investigation contributes to a better understanding of the molecular differences associated with a TEM and may offer insights into potential therapeutic strategies, as well as directions for future research. A key limitation of the study is the small sample size. Additionally, hysteroscopy the gold standard for diagnosing intrauterine lesions was not employed; instead, SIS was used due to its lower invasiveness and cost. Notably, previous studies have demonstrated that SIS offers comparable diagnostic accuracy to hysteroscopy for identifying intrauterine abnormalities such as adhesions, polyps, or submucous myomas (Cepni *et al.*, 2005; Seshadri *et al.*, 2015). Gene expression analysis was conducted during the late proliferative phase under COS, which may limit the generalizability of the findings to natural or hormone replacement therapy (HRT) cycles. Future studies should explore the molecular mechanisms underlying TEM in various endometrial preparation protocols to inform novel therapeutic strategies aimed at enhancing endometrial receptivity and improving IVF outcomes.

## CONCLUSION

In summary, this study explored gene expression differences between thin endometrium and normal endometrium in women undergoing controlled ovarian stimulation. The level of *SUSD2* expression, a marker for endometrial stem/progenitor cells in the perivascular area, seems to be related to endometrial growth retardation. Although no significant differences in gene expression were observed in the stromal and epithelial compartments, our findings suggest that serum estradiol levels may influence gene expression across different endometrial components. These insights may provide clues for future treatments targeting thin endometrium.
